# Type 2 Salter-Harris Physeal Injury of the Proximal Phalanx of Great Toe: A Case Report and Review of Literature

**DOI:** 10.7759/cureus.16272

**Published:** 2021-07-08

**Authors:** Vivek Tiwari, Samir Dwidmuthe, Samrat S Sahoo

**Affiliations:** 1 Orthopaedics, All India Institute of Medical Sciences Nagpur, Nagpur, IND

**Keywords:** physeal injury, proximal phalanx, great toe, salter-harris classification, foot injury

## Abstract

Fractures involving the physes are peculiar to the pediatric age group with growing bones. These injuries, if not managed with due precaution, can lead to complications like physeal arrest and the development of deformity. Such injuries are much more common in the upper limbs as compared to the lower limbs. Physeal injuries involving the small bones of the foot are extremely rare. To date, no case of Salter-Harris type 2 physeal injury involving proximal phalanx of the great toe has been reported in the English literature. We report the first such case of a 10-year-old child who sustained a Salter-Harris type 2 physeal injury of the right great toe proximal phalanx. The fracture was managed conservatively with splintage of the great toe, and the child had good outcome at one year without any adverse sequelae.

## Introduction

Physeal injuries are unique injuries limited to the pediatric age group. As the cartilaginous physeal region is less strong and often less resistant to the shear and tension forces than the adjacent bony and fibrous tissues, any traumatic force to that region dissipates through the physis [1]. They constitute nearly one-fifth of all pediatric age orthopedic injuries [2-5]. Such injuries, if not managed properly, can have long-term sequelae in the form of growth arrest and deformity. The factors that can affect the outcome after these injuries include the age of the patient, the pattern of injury, its location, whether it was an open fracture or closed injury, status of the surrounding soft tissues, and epiphyseal blood supply [6]. Salter and Harris proposed a prognostic classification of physeal injuries in 1963 consisting of five types of injuries [7]. The majority of Salter-Harris type 1 and 2 injuries heal well with conservative management, whereas type 3 may require operative intervention, and type 4 injuries almost always need surgery.

Although physeal injuries predominantly involve upper limbs after a history of a fall on an outstretched hand, other parts of the body including the foot can also get affected. However, there have been very sparse reports of physeal injuries involving the foot bones [8]. Moreover, physeal injuries of the phalanges of the foot are extremely rare [5,9-11]. To our knowledge, there has been no report in the English literature of a Salter-Harris type 2 physeal injury of the great toe proximal phalanx. We report one such unique case of a 10-year-old boy presenting with a Salter-Harris type 2 physeal injury involving the proximal phalanx of the great toe, which was successfully managed conservatively, without any adverse sequelae at one-year follow-up.

## Case presentation

**Figure 1:**
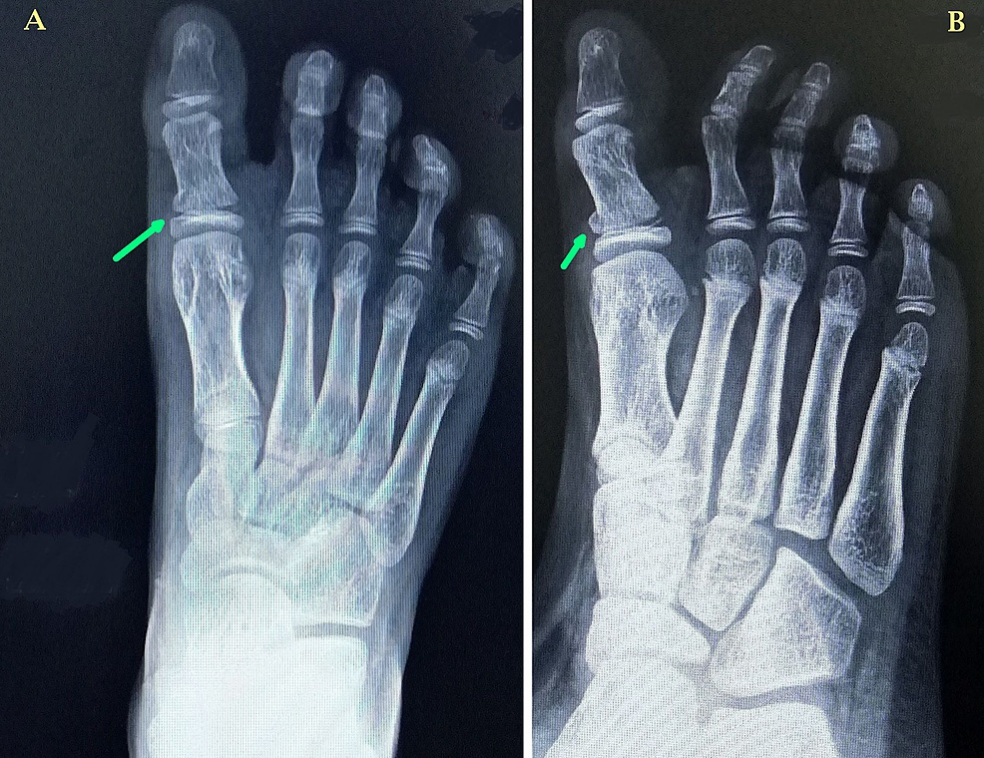
Plain radiographs of the right foot – anteroposterior (A) and oblique (B) views. The radiographs demonstrate a fracture in the base of the proximal phalanx of the great toe with a metaphyseal fragment (green arrows) making it a Salter-Harris type 2 physeal injury.

A 10-year-old boy presented to the orthopedics outpatient department of our institute with right great toe pain and swelling after having snubbed into a wall while playing. The injury occurred one day back. At presentation, the child was not able to move his great toe because of severe pain and discomfort. There was no other concomitant injury and his medical history did not reveal any abnormality. The child’s general physical and systemic examination was unremarkable. On local examination, there was severe tenderness and mild swelling over the first metatarsophalangeal joint of the right foot. There was no visible deformity of the great toe. Passive flexion-extension movements of the great toe were extremely painful. The overlying skin and distal neurovascular examinations were normal. Plain radiographs of the right foot were done, which revealed a fracture in the base of proximal phalanx of the great toe with a metaphyseal fragment, making it a Salter-Harris type 2 physeal injury (Figure 1).

**Figure 2:**
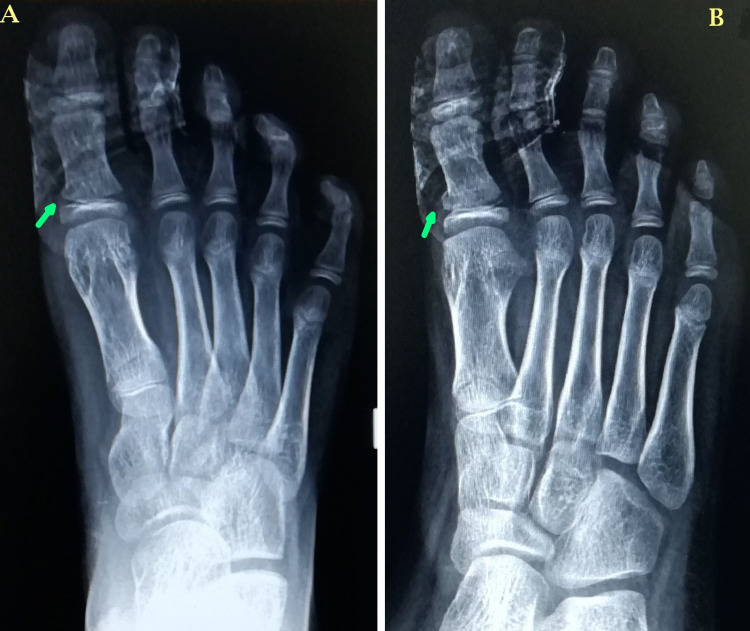
One-week follow-up plain radiographs of the right foot – anteroposterior (A) and oblique (B) views. The plain radiographs show acceptable alignment of the fracture (green arrows) and great toe at one week after splinting of the great toe with second toe.

Under sedation, longitudinal traction was applied, and splinting of the great toe was done with the second toe. Also, a below-knee splint was provided for support. At one-week follow-up, pain and swelling were reduced and the radiographs showed acceptable alignment of the fracture and great toe after splinting (Figure 2).

**Figure 3:**
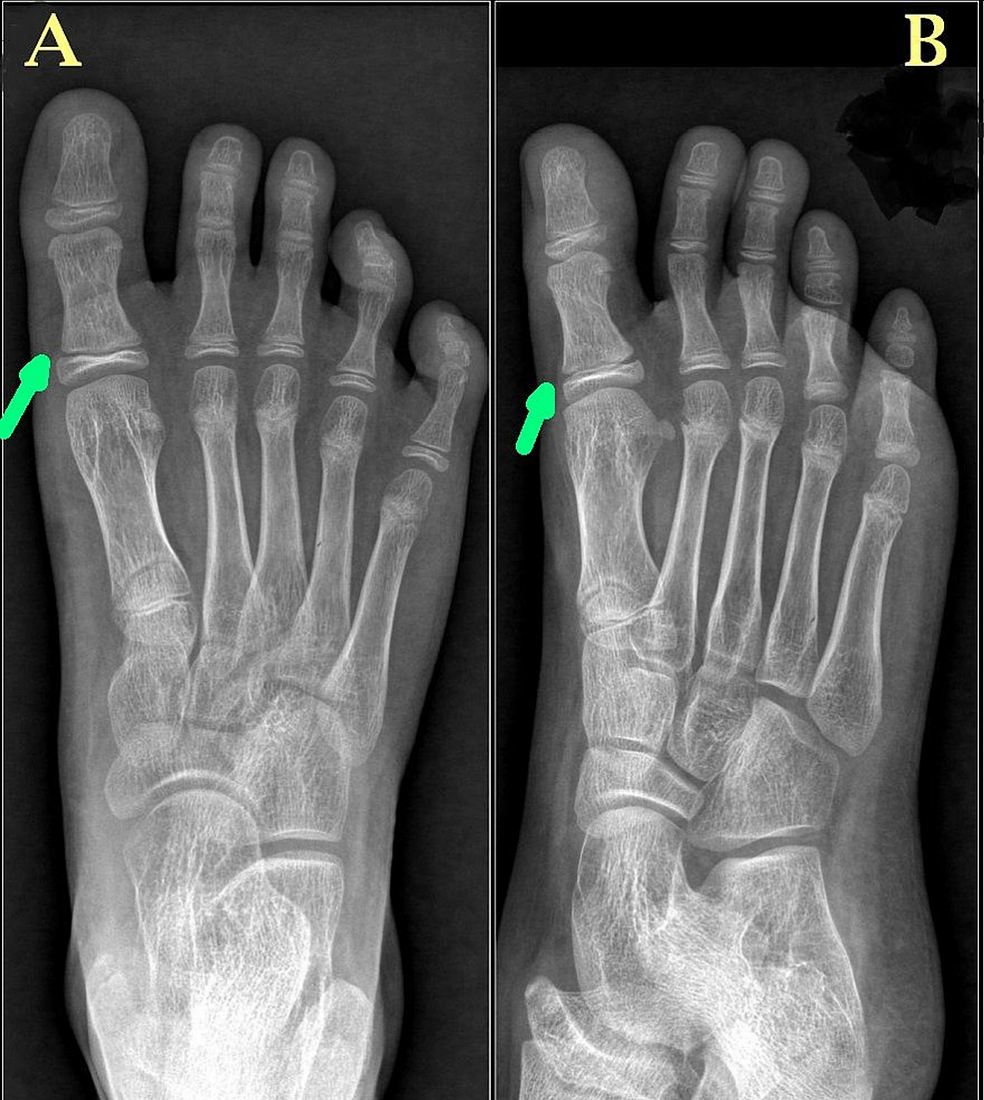
One-year follow-up plain radiographs of the right foot – anteroposterior (A) and oblique (B) views. The plain radiographs show complete healing of the fracture (green arrows) with normal  alignment of the great toe without any signs of physeal irregularity or growth arrest.

The child’s parents were advised to keep the great toe splintage for three weeks. However, the patient was lost to follow-up. The child later presented after one year in the outpatient department. At the last follow-up of one year, the child was completely pain-free with full weight-bearing. The alignment of the right great toe was normal, and both the great toes were of similar length. The follow-up plain radiograph showed complete healing of the fracture with normal alignment of the great toe without any signs of physeal irregularity or growth arrest (Figure 3).

## Discussion

Physeal injuries are more frequently seen these days due to the increased involvement of children in sports activities [1]. However, upper limb injuries are more frequently encountered than lower limb injuries [12]. Moreover, such injuries in small bones of the hand and feet are relatively less discussed and reported in the literature than the long bone fractures [8]. Fractures involving the foot phalanges in children are quite rare and occur chiefly as a result of low-energy trauma like snubbing of the foot against a wall while playing or kicking an object [13]. Physeal injuries involving the great toe phalanges are extremely rare.

Salter-Harris type 2 injuries are the most commonly encountered physeal injuries overall, accounting for nearly 70% of all such injuries [14]. The prognosis of such injuries is good, and the majority of such fractures can be successfully managed conservatively in a splint. However, closed reduction should be done with a single careful manipulation in order to prevent growth plate damage. On the other hand, Salter-Harris type 4 fractures generally require open reduction and internal fixation and are prone to developing complications like malunion and growth arrest if not properly addressed [4,7]. All such patients with physeal injuries should be followed up to look for any physeal arrest and development of deformity. Such injuries involving the great toe, if not managed properly, can lead to problems with ambulation, shoe wear, as well as participation in athletic activities.

We report the first case in the English literature of a Salter-Harris type 2 physeal injury involving the great toe proximal phalanx, managed conservatively with a good outcome, though Salter-Harris type 2 fractures have been reported in the other toe phalanges [5,9]. Oliva et al. reported type 2 fracture in the fifth toe proximal phalanx in a three-year-old girl, managed conservatively without any sequelae [5]. Similarly, Murdock et al. described a type 2 physeal injury in second toe proximal phalanx in a nine-year-old child managed conservatively with good results [9].

Salter-Harris types 3 and 4 fractures have been described in the great toe proximal phalanx [10,11]. Buch et al. reported a case of Salter-Harris type 4 injury of proximal phalanx great toe, which was managed with open reduction and K-wire fixation with good functional outcomes at one year [10]. Mafulli described two cases of Salter-Harris type 3 injury of proximal phalanx great toe [11]. In the above series, one case required open reduction and K-wire fixation and had a good outcome at four years of follow-up, and the other case was managed conservatively in a soft bulky bandage and had good results at three years of follow-up. Neither of the above patients had any physeal arrest/deformity/length discrepancy. Our patient with type 2 injury also did not show any such long-term complications at the last follow-up of one year.

## Conclusions

This case reports Salter-Harris type 2 physeal injury of the proximal phalanx great toe in a 10-year-old child. To date, no such case has been reported in the English literature. These types of injuries can be successfully managed conservatively, as done in our case, with good outcomes, and without any adverse sequelae.
